# Editorial: Genetics of Kidney Diseases

**DOI:** 10.3389/fgene.2020.00305

**Published:** 2020-04-03

**Authors:** Martin H. de Borst, Harvest F. Gu

**Affiliations:** ^1^Division of Nephrology, Department of Internal Medicine, University Medical Center Groningen, University of Groningen, Groningen, Netherlands; ^2^Center for Pathophysiology, School of Basic Medicine and Clinical Pharmacy, China Pharmaceutical University, Nanjing, China

**Keywords:** chronic kidney diseases, diabetic kidney disease, genome-wide association study, kidney transplantation, next-generation sequencing, precision medicine

Worldwide, more than 850 million people have chronic kidney disease (CKD) (Jager et al., 2019). CKD can be caused by a variety of individual diseases including primary kidney diseases and systemic diseases such as diabetes. Diabetic kidney disease (DKD) worldwide develops in ~40% of patients with diabetes and is the leading cause of CKD (Alicic et al., 2017). CKD is accompanied by an excessively elevated risk of premature mortality, particularly due to its predisposition to accelerated cardiovascular disease. In more than 10% of CKD patients the primary cause of disease is unknown (Groopman et al., 2018). It is crucial to increase the yield of the diagnostic work-up in these patients, since specific treatments exist for several kidney diseases.

Recent developments in genetics including next-generation sequencing (NGS) have strongly enhanced the diagnostic potential for patients with CKD. Moreover, novel genetic tools have contributed to growing insight in the etiology of both monogenic and multifactorial types of CKD. This is strongly illustrated by the rare disease atypical hemolytic uremic syndrome (aHUS), for which now numerous causal mutations in complement-related genes have been identified (Jokiranta, 2017). In this Research Topic, Schönauer et al. have demonstrated the case of a patient with aHUS, which was caused by a novel splice site variant in the complement factor H gene. Another paper in this Research Topic (Li P. et al.) also reports on a splice site mutation, in this case in the alpha-galactosidase A (*GLA*) gene. Several mutations in this gene have been previously found that lead to Fabry disease, a rare X-linked recessive hereditary systemic disorder of glycosphingolipid metabolism caused by totally or partially decreased activity of *GLA* (Simonetta et al., 2018). The aforementioned studies illustrate the power of genetic tools to reach a clinically relevant diagnosis driving the CKD phenotype (although the disorder may also affect other organ system as for example in Fabry disease), which may have therapeutic consequences as specific treatments exist both for aHUS and Fabry.

At the same time, clinical practice in nephrology is facing new challenges including optimal patient selection, implementation, counseling, and therapeutic consequences of the outcomes of NGS-based diagnostics. De Haan et al. discuss the advantages and limitations of NGS-based tools, and specifically focus on how these tools could improve diagnostic yield in patients with CKD of unknown or unclear etiology. In fact, further prospective cohort studies are needed to define the optimal positioning of NGS-based genetic testing in the diagnostic workup of CKD with unknown etiology.

For multifactorial diseases, which affect the majority of CKD patients, the impact of the “genomic revolution” has so far been relatively limited. At the same time these advances, among others in terms of technology and infrastructure, have the potential to revolutionize precision medicine in the field of nephrology. This is illustrated by International Genetics & Translational Research in Transplantation Network (iGeneTRAiN): a multi-site consortium that encompasses >45 genetic studies with genome-wide genotyping from over 51,000 transplant samples, including genome-wide data from >30 kidney transplant cohorts (*n* = 28,015) (Fishman et al.). The potential of large-scale collaborations such as iGeneTrain to contribute to the understanding of disease etiologies has already been shown in several studies in CKD and kidney transplantation (Snoek et al., 2018; Reindl-Schwaighofer et al., 2019). Furthermore, genome-wide association studies (GWAS) performed in diverse populations can be useful to define the robustness of previously identified CKD risk loci such as the *APOL1* across different ethnicities. This multi-ethnic study by Lin et al. also identified a novel risk locus for CKD near the *NMT2* gene. Yet another approach is to study very specific high-risk populations. Thomson et al. performed a GWAS of limited size in a cohort of Australian Aboriginal Tiwi islanders, a population prone to develop CKD that had not been extensively studied before. The authors identified a variant near the *CRIM1* gene that was associated with albuminuria, and remained significant after adjusting for multiple testing (Thomson et al.).

Another important application of genetic tools is in patient stratification, and in the identification of modifier genes that enhance susceptibility to morbidities such as CKD and DKD. This particularly applies to patients with type 1 and type 2 diabetes, the most common cause of end-stage kidney disease (ESRD) worldwide (Gu). Valoti et al. identified a variant in the complement factor H (CFH) gene that confer patients with type 2 diabetes at increased risk of microalbuminuria and cardiovascular complications. Moreover, patients carrying the variant were less likely to benefit from ACEi therapy. Although CFH mutations are well-known to predispose to aHUS (Jokiranta, 2017), it was so far not known that CFH variants could enhance susceptibility to adverse kidney outcomes in patients with diabetes. Two variants in the beta-actin (*ACTB*) gene were identified in another study, which were associated with a higher risk of DKD in a large cohort of patients with type 2 diabetes (Li M. et al.). Thereby, *ACTB*, as a housekeeping gene, is suggested preferably not to be used as internal control for gene expression studies at the mRNA and protein levels in diabetes and DKD.

Despite efforts summarized above to find missing pieces of the genetic puzzle in CKD and DKD, it remains challenging to explain the complete heritability with currently available methods and datasets. Although studies beyond “conventional GWAS” have focused on telomeres, copy number variants, mitochondrial DNA and sex chromosomes, there remains considerable unexplained heritability in CKD and DKD (Cañadas-Garre et al.).

Furthermore, in addition to the multitude of information coming to us from genetic studies, we are facing new challenges as these data need to be interpreted in the context of other data dimensions including proteomics, single-cell RNA-sequencing, metabolomics, and the microbiome (Parsa et al., 2013; Hanna et al., 2017). Therefore, in order to profoundly impact clinical practice, comprehensive approaches are needed, particularly through the integration of multiple-omics data.

## Author Contributions

MB and HG co-edited the Research Topic, wrote, edited, and approved the final version of the Editorial.

### Conflict of Interest

The authors declare that the research was conducted in the absence of any commercial or financial relationships that could be construed as a potential conflict of interest.
